# The Genetics of Dementia with Lewy Bodies: Current Understanding and Future Directions

**DOI:** 10.1007/s11910-018-0874-y

**Published:** 2018-08-10

**Authors:** Tatiana Orme, Rita Guerreiro, Jose Bras

**Affiliations:** 1 Department of Neurodegenerative Disease, UCL Institute of Neurology, London, UK; 2 UK Dementia Research Institute at UCL, Institute of Neurology, Wing 1.2, The Cruciform Building, Gower Street, London, WC1E 6BT UK; 3 Department of Medical Sciences and Institute of Biomedicine, iBiMED, University of Aveiro, 3810-193 Aveiro, Portugal

**Keywords:** Dementia with Lewy bodies, DLB, Genetics, GWAS, Next-generation sequencing

## Abstract

**Purpose of Review:**

Dementia with Lewy bodies (DLB) is a neurodegenerative disease that can be clinically and pathologically similar to Parkinson’s disease (PD) and Alzheimer’s disease (AD). Current understanding of DLB genetics is insufficient and has been limited by sample size and difficulty in diagnosis. The first genome-wide association study (GWAS) in DLB was performed in 2017; a time at which the post-GWAS era has been reached in many diseases.

**Recent Findings:**

DLB shares risk loci with AD, in the *APOE* E4 allele, and with PD, in variation at *GBA* and *SNCA*. Interestingly, the GWAS suggested that DLB may also have genetic risk factors that are distinct from those in AD and PD.

**Summary:**

Although off to a slow start, recent studies have reinvigorated the field of DLB genetics and these results enable us to start to have a more complete understanding of the genetic architecture of this disease.

## Introduction

Dementia with Lewy bodies (DLB) is a neurodegenerative disease that shares clinical, pathological and genetic features with Parkinson’s disease (PD) and Alzheimer’s disease (AD). Lewy body dementia is a term that encompasses both DLB and Parkinson’s disease dementia (PDD), and the ‘one year rule’ is used to assess the temporal onset of dementia versus parkinsonism, in order to differentiate the two diseases. If parkinsonism occurs at the same time or within 1 year of dementia, a diagnosis of DLB is made, whereas if parkinsonism precedes the onset of dementia by a year or more, PDD is diagnosed. The fundamental feature of DLB is dementia, which often occurs with parkinsonism, early fluctuations in cognition and attention, visual hallucinations and REM sleep behaviour disorder (RBD) [1••, 2]. Autonomic dysfunction, sleep irregularities, depression and anxiety are also common. DLB is a devastating disease because of its symptoms, as well as the fact that there is currently no cure or disease modifying therapy available. Furthermore, DLB is thought to have a shorter disease duration [3] and decreased survival rate compared to AD [4]. The need to understand the disease pathobiology is imperative for the development of disease-specific therapies.

It is now clear that DLB has a strong genetic component. Although most cases are sporadic, a number of reports have demonstrated the occurrence of the disorder in families [5–13], in addition to the identification of genetic loci that modulate risk for the development of DLB [14, 15, 16••]. As such, using common genetic variants, the proportion of the phenotype that can be attributed to genetic factors was estimated to be 36% [16••].

## DLB Genetics Has Remained Largely Elusive

Despite the fact that we now know that genetics plays a role in the disease, genes that cause DLB are still to be identified. In comparison with AD and PD, we know far less about the genetic basis of DLB. There are multiple reasons for this. Firstly, DLB is difficult to diagnose, as phenotypic overlaps with other neurodegenerative diseases can diminish chances of an accurate diagnosis. The lines between different neurodegenerative diseases can often be blurred, and a patient may show features of one or several other diseases, both ante and post mortem. Additionally, DLB is not as frequently recognized as a disease compared to other well-known disorders, such as AD. Both factors result in a substantial rate of mis- and under-diagnosis. This has hindered the collection of large cohorts of cases whose diagnosis is certain, and as a consequence, has limited large-scale genetic analyses. Furthermore, as the disease is age-related, and typically occurs in those aged 65 or older, the likelihood of gathering biomaterials from multiple family members for genetic testing of familial DLB is small. This, coupled with the fact that families with DLB are rare, has limited the understanding of Mendelian DLB genetics. Although still a prevalent cause of disease in the elderly, DLB is less common than diseases such as AD and PD, and hence cohorts will generally be smaller for this disease.

**Fig. 1:**
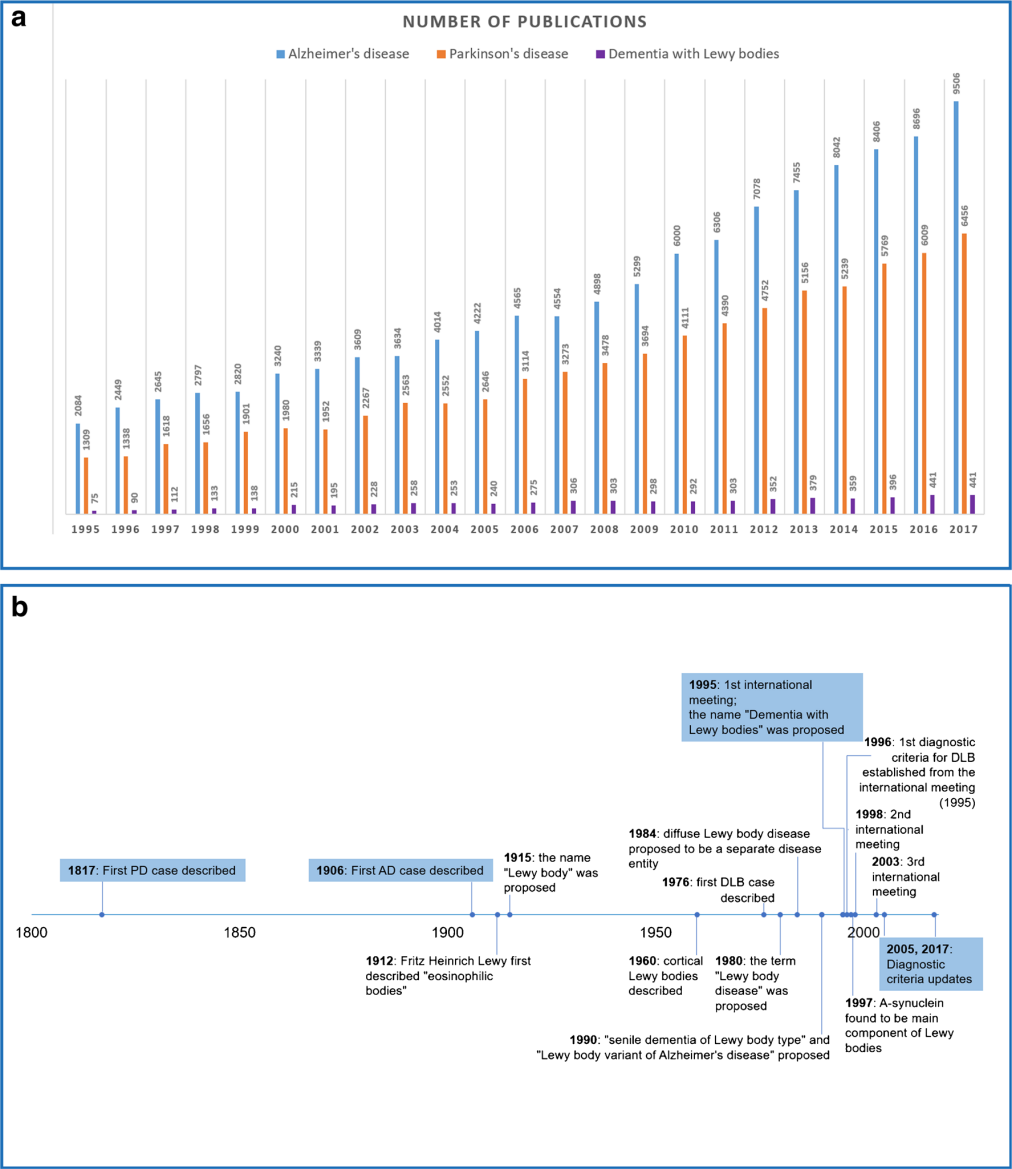
Number of publications and important landmarks in Dementia with Lewy bodies. **a** Number of research publications in Alzheimer’s disease, Parkinson’s disease and dementia with Lewy bodies from 1995 to 2017. Research publications are far fewer in dementia with Lewy bodies as compared to Alzheimer’s or Parkinson’s diseases. The terms ‘Alzheimer’s disease’, ‘Parkinson’s disease’ and ‘Dementia with Lewy bodies’ were used as input for PubMed and the resulting number of publications per year were plotted. The time points begin in 1995, when DLB was first established as a disease entity. **b** Timeline of important landmarks in dementia with Lewy bodies. Parkinson’s and Alzheimer’s disease were proposed as disease entities many years before the establishment of DLB as a disease (1817, 1906 versus 1976); however, cortical Lewy bodies were identified in 1960. The first diagnostic criteria for DLB were established in 1996, and updated in 2005 and 2017. This timeline has been created using the historical landmarks for DLB from Kenji Kosaka’s chapter (chapter 1), in his recent book [33]. PD = Parkinson’s disease, AD = Alzheimer’s disease, DLB = dementia with Lewy bodies

Moreover, some previous genetic studies have focused on Lewy body disease (PD, DLB and PDD) as a whole, and not specifically to genetic alterations that are unique to DLB (Table 1). Ultimately, there is less research conducted in DLB compared to AD and PD (Fig. 1a). DLB was only described as a separate disease entity in 1984, comparatively later than AD or PD (Fig. 1b). Thus, genetic research in DLB is only just gathering momentum; for example, the first genome-wide association study (GWAS) in DLB was conducted in 2017 [16••], at a time when we have reached the post-GWAS era in many diseases. Genetic studies have already shown that DLB shares genetic risk factors with AD and PD; however, recent findings suggest that DLB may also have a unique genetic architecture [16••].

**Table 1:** Representative examples of genetic studies conducted in sporadic DLB

Study type	Gene(s) or variant analysed	Ethnicity, population	Cases	Controls	Clinically or pathologically diagnosed DLB	DLB diagnostic criteria	Main finding	Ref.
Candidate gene	*C9orf72*	Caucasian—North American	111 DLB*	0	All with pathological diagnosis—86 neocortical, 25 transitional. 66% met pathological criteria for AD	2005	No expansions > 30 repeats found	[17]
Candidate gene	*C9orf72*	Caucasian—UK	102 DLB	0	All with clinical diagnosis—probable DLB	2005	2 clinical DLB with > 30 repeats	[18]
Candidate gene	*C9orf72*	Caucasian—European, American, Australian	1524 DLB***	0	1398 pathologically, 126 clinically diagnosed	2005	*C9orf72* repeat expansions not common in pathologically diagnosed DLB	[19]
Candidate gene	*ADORA1* sequencing	Caucasian—North American	111 DLB* and 1214 PD cases	4911	All DLB pathologically diagnosed—86 neocortical, 25 transitional	2005	*ADORA1* variants not common in PD or DLB	[20]
Candidate gene	Exon 24 of *DNAJC13*	Caucasian—US, European	1938 PD, 828 LBD	0	1938 clinical PD, 828 pathologically diagnosed LBD	2005	Did not find p.Asn855Ser in any cases	[21]
Candidate gene	*TREM2* p.Arg47His	Caucasian—American	1271 total LBD	1154	442 clinical DLB cases, 829 pathologically diagnosed LBD cases: high (349), intermediate (254), and low clinical DLB likelihood (226)	2005	p.Arg47His not associated with DLB	[22]
Candidate gene	*RAB39B* sequencing	Caucasian—American	884 PD, 399 DLB and 379 LBD**	0	Clinical DLB, pathologically diagnosed LBD	Unclear, 2005 (?)	No coding variants found	[23]
Candidate gene	*MAPT* haplotype genotyping	Caucasian—American	731 DLB**	1049	431 clinically diagnosed, 347 pathologically diagnosed (high-likelihood)	Clinical 2005 1996, pathological - 2005	*MAPT* H1G haplotype suggested to be associated with DLB	[24]
Candidate gene	*MAPT* p.Ala152Thr	Caucasian —American, European	3229 PD, 442 DLB, 181 MSA and 832 LBD**	2456	All clinical DLB	2005	p.Ala152Thr suggested to be associated with DLB and LBD	[25]
Candidate gene	Certain *LRRK2* variants	Caucasian—American	725 total DLB**	1790	417 clinical DLB (384 probable DLB, 33 possible DLB), 355 pathologically diagnosed high likelihood DLB. (47 cases in both)	2005	No significantly associated *LRRK2* variants with DLB	[26]
Candidate genes (multiple)	*GBA, LRRK2, MAPT, APOE, APP, PSEN1, PSEN2, SCARB2* and *SNCA*	Caucasian—North American	111 DLB*	222 neuro normal	All pathologically diagnosed—86 neocortical, 25 transitional 69% also met pathological criteria for AD	2005	Several variants identified	[27]
Candidate genes (multiple)	PD and AD loci	Caucasian—European, American, Australian	788 DLB***	2624	667 pathologically diagnosed	2005	*SNCA*, *APOE* significantly associated with DLB, whilst. *SCARB2* showed suggestive association	[28]
Candidate genes (multiple)	CNV analysis: *APP*, *SNCA*, *PARK2*. Selected exons: *APP*, *LRRK2*. Majority of exons: *PSEN1*, *PSEN2*, *MAPT*, *GRN*, *TARBP*, *SNCA*, *PARK2*, *PINK1*, *DJ-1*, *APOE* and *GBA*	Caucasian—Belgian	99 DLB and 75 PDD	626	Majority clinically diagnosed	2005	Several variants identified	[29]
Candidate genes (multiple)	*SNCA, LRRK2, UCHL1, GIGYF2, Omi/HTRA2, EIF4G1, PARK2, PINK1, ATP13A2, PLA2G6, FBX07, DJ-1, APP, PSEN1, PSEN2, C9orf72, SOD1, MAPT, PGRN, TARDBP, OPTN, ANG, CHMP2B, SQSTM1, FUS, VCP, OPTN*	Not reported, likely Caucasian—UK	91 DLB	93	All pathologically diagnosed	2005	Several variants identified	[30]
Candidate genes (several)	43 tagging SNPs at the *SNCA* locus, *SNCA* dosage*, APOE* genotype	Caucasian—European, North American	1492 PD and 922 DLB	971	518/922 DLB pathologically diagnosed	2005	Dementia associated 5′ parkinsonism associated 3′ of *SNCA*	[31]
Genetic analysis	Exome sequencing, *APOE* genotyping, *C9ORF72* repeat analysis, CNV analysis	Likely Caucasian, not confirmed—UK	289 AD, 252 FTD/ALS, 239 CJD, 39 PD, 58 DLB, 266 other neurodegenerative disease, 368 controls	266 brains, 380 total controls used for association analysis	All DLB pathologically diagnosed	2005	*TREM2* p.Arg62His and *GRN* rare variants suggested to be associated with DLB	[32]
GWAS	Genome-wide genotyping	Caucasian—European, North American, Australian	1743 DLB***	4454	1324 pathologically diagnosed, intermediate to high likelihood of DLB	2005	*SNCA, APOE, GBA* were genome-wide significantly associated with DLB	[16••]

Genetic studies in DLB are limited compared to Alzheimer’s and Parkinson’s disease. Furthermore, most genetic studies in DLB are focused on one or more candidate genes, highlighting the need for an unbiased, genome or exome-wide view of DLB genetics. Where possible to ascertain, DLB patients that are included in multiple studies are denoted by *, **, or ***. Some DLB patients may have a family history of disease; however, the majority of analysis focused on sporadic patients and not DLB families. Genetic studies in families with DLB phenotypes have previously been reviewed [85]. Studies solely investigating *APOE* and *GBA* are not included in the table but are discussed in the main text. A mixture of clinical and pathologically diagnosed DLB patients are common in genetic studies. Some studies combine PD, PDD and DLB, or PDD and DLB patients into one study group, which negates identification of DLB specific variants

*Ref* reference, *GWAS* genome-wide association study, *PD* Parkinson’s disease, *AD* Alzheimer’s disease, *DLB* dementia with Lewy bodies, *LBD* Lewy body disease, *PDD* Parkinson’s disease dementia, *FTD/ALS* frontotemporal dementia/amyotrophic lateral sclerosis, *CJD* Creutzfeldt Jakob disease, *MSA* multiple system atrophy, *CNV* copy number variation

## Well-established Genetic Findings in DLB: *APOE, SNCA* and *GBA*

In order to adequately implicate a gene in disease, detailed assessment of the gene in question is required, with a view to reproduce findings in independent studies. Replication studies are fundamental to distinguish true associations from false positive findings, and thus in the validation of the results of the initial study [34]. To date, only three genes have been convincingly established to be involved in DLB: *APOE*, *GBA* and *SNCA*. Variation in the *SNCA* gene can modulate risk for or cause DLB phenotypes. The established risk factors for DLB are also known to impart risk to either AD (*APOE*) or PD (*GBA*, *SNCA*). Of note, AD and PD do not share common genetic risk factors between them [35]. This is in accordance with a recent study, which showed no evidence of correlation between PD and AD, but showed that the genetic correlation between DLB and AD was 0.578 (SE ± 0.075), and between DLB and PD was 0.362 (SE ± 0.107). The *APOE* locus is highly associated with AD and DLB. When removing this locus from the analysis, the correlation between AD and DLB was reduced to 0.332, which does not differ significantly from the correlation between DLB and PD [36]. It should be noted that this study used a genotyping array enriched for neurological disease variants and so was not entirely unbiased.

### SNCA

The *SNCA* gene was first implicated in DLB when point mutations, p.Ala53Thr and p.Glu46Lys, and locus multiplications were identified in families with mixed phenotypes of parkinsonism and dementia that resembled DLB [37–39]. Locus multiplications resulting in three copies (heterozygous duplication) or four copies (homozygous duplication or heterozygous triplication) of *SNCA* have been described in PD, PDD and DLB [39–44], and a comprehensive review of *SNCA* mutations in parkinsonism was conducted by Rosborough and colleagues [45]. Pathogenic mutations in *SNCA* are very rare, can result in a wide phenotypic spectrum from PD, PDD, DLB, multiple system atrophy (MSA) and even frontotemporal dementia (FTD) [46–49], and can show heterogeneity between members of the same family in terms of age of onset, phenotype and pathology. Furthermore, not all duplications are fully penetrant [41]. *SNCA* disease-causing point mutations fall in the amphipathic region of alpha-synuclein; however, their role in disease is not clear, as not all mutations have the same effect. It has been hypothesized that these mutations may perturb membrane binding activity, or initiate disease by increasing the propensity of the protein to aggregate. Triplications result in overexpression of SNCA mRNA and protein [50], causing a more severe phenotype and earlier disease onset.

In addition to causing disease, *SNCA* has also been shown to modulate disease risk for DLB and PD. Interestingly, PD and DLB show differential association profiles at the *SNCA* locus, a phenomenon first reported in a study analysing AD and PD-associated loci in DLB [28]. This difference was also observed in the recent DLB GWAS (which had some overlap of samples included in the previous study) [16••]. In detail, the most associated single nucleotide polymorphism (SNP) at the *SNCA* locus in a meta-analysis of GWA studies in Parkinson’s disease is found 3′ to the gene (rs356182) [51]; this SNP was not significant in the DLB GWAS, where instead, association at the *SNCA* locus is mediated by a SNP 5′ of the gene (rs7681440). Guella and colleagues also found in a study of DLB, PD, PDD and healthy controls that the risk for dementia was associated with a locus located 5′ of the gene, and the risk for parkinsonism was associated with variants located 3′ to the gene [31]. Using GTEx data, it was shown that the rs7681440 SNP is an eQTL for RP11-67M1.1, an antisense gene and a negative regulator of *SNCA* expression. The alternative allele mediates an increase in *SNCA* expression in the cerebellum through decreasing expression of the SNCA repressor [16••], which would fit with a mechanism of increased *SNCA* expression increasing risk for disease, although further studies are required to confirm this hypothesis.

Interestingly, the most significantly associated SNP in *SNCA* in DLB was shown to be in linkage disequilibrium (LD) with a SNP in PD that was significantly associated with the disease once the most significant SNP was removed [51].

*SNCA* methylation was suggested to be significantly decreased in DLB [52], albeit in a small study of 20 clinical DLB cases and 20 controls. Differential expression of *SNCA* isoforms in DLB have been proposed [52, 53], but requires further study due to small sample sizes. A systematic review concluded that DLB patients have decreased alpha-synuclein in CSF and this may be used to differentiate these patients from AD, but not PD cases [54]**.**

REP1 is a polymorphic microsatellite repeat upstream of the *SNCA* translation start site. Variations in REP1 length have been associated with PD [55], and PD-associated REP1 polymorphisms enhance *SNCA* transcription in transgenic mice [56]. It was later shown that a SNP in *SNCA* identified to be associated with PD in a GWAS (rs3857059) is in LD with the REP1 risk allele (*D*′ = 0.872, *R*^2^ = 0.365) [57]; however, in a separate, albeit small study, REP1 was not associated with PD [31]. So far, it is not known whether REP1 polymorphisms are involved in DLB, given that this has not been tested specifically.

The *SNCA* gene is highly relevant to synucleinopathies as its encoded protein, alpha-synuclein, aggregates within neurons to form Lewy bodies and Lewy neurites [58], which are the pathological hallmark of PD, PDD and DLB. The anatomical distribution of Lewy-related pathology is often widespread to limbic and neocortical areas in DLB and PDD, and at end stage, the diseases are indistinguishable. The presynaptic aggregation of alpha-synuclein is thought to cause neurodegeneration in DLB [59], where it has been shown to be phosphorylated [60]. Alpha-synuclein aggregation in neurodegenerative diseases not only differs in the brain regions affected, but also the cell types in which it is found, for example, the aggregation of alpha-synuclein in glial cytoplasmic inclusions (GCI) is typically seen in multiple system atrophy (MSA). A recent study demonstrated that the alpha-synuclein species in GCIs and LBs are conformationally and biologically distinct [61•]. Lewy-related pathology can also been seen as a secondary pathology in Alzheimer’s disease [62], predominantly in the amygdala [63].

### *APOE*

Genetic variation at two SNPs (rs429358 and rs7412) in the *APOE* gene result in three alleles, of which the ε4 allele is well established to increase the risk of developing Alzheimer’s disease in a dose-dependent manner [64, 65]. *APOE* ε4 allele dosage has also been shown to be a risk factor for the development of DLB, and whilst first identified over 20 years ago [14], has since been replicated in a number of studies [3, 16••, 27–30, 66].The protective effect of the ε2 allele is less well established in DLB, conferring protection in some studies [67] but not others [3]. DLB patients who carry ε4 alleles die at a younger age than those who do not [30]. It was hypothesized that the association of *APOE* ε4 in DLB may be driven by the presence of AD-related neuropathology [68], which can often be seen alongside Lewy-related pathology in DLB brains. However, the association with *APOE* ε4 remains robust in ‘pure’ DLB cases who have Lewy bodies but minimal or low-level Alzheimer-related pathology [66], perhaps suggesting that *APOE* is correlated to dementia in a mechanism unrelated to the amyloid cascade. Interestingly, *APOE* is not a risk factor for PD [69], which demonstrates its specificity for dementia risk. Furthermore, a recent study showed that *APOE* ε4 dose was associated with decreased hippocampal volumes irrespective of AD or DLB diagnosis [70].

*APOE* encodes apolipoprotein E, a protein that is involved in lipid binding, transport and receptor-mediated endocytosis [71] and that is suggested to influence AD pathogenesis through mechanisms related to amyloid beta (Aβ) aggregation and clearance. However, other possible pathological processes involving tau phosphorylation, neuroplasticity and neuroinflammation have been proposed [72].

*APOE* alleles differ in CpG content and in ε3/ε4 genotype carriers, *APOE* CpG island methylation was significantly decreased in the frontal lobe of AD and DLB patients, with the most significant decrease in DLB patients with mixed AD and LB pathology, as compared to pure AD or LB pathology cases [73]. Although further studies are required to replicate this finding, and to analyse differential methylation of other *APOE* genotypes in DLB, this may suggest that epigenetic changes at this locus play a role in disease etiology.

### *GBA*

Homozygous mutations in the *GBA* gene cause Gaucher disease (GD); through astute clinical observation, it was noted that some GD patients showed parkinsonian features [74], and that heterozygous carriers of these variants had a higher prevalence of PD [75], leading to the discovery that heterozygous variants in *GBA* can predispose to PD, with an odds ratio (OR) of 5.43 (95% CI, 3.89–7.57) [76]. This was also shown to be true for DLB [15, 16••, 27, 30, 77–83], with an OR of 8.28 (95% CI, 4.78–14.88), where *GBA* variants are linked to earlier disease onset and death [15].

PD patients with *GBA* variants have an approximate 2.4-fold increased incidence of cognitive impairment [84] and, given the increased OR for DLB, suggests a strong affinity of *GBA* variants for Lewy body dementia. Whilst a role for variants in *GBA* in DLB disease risk modulation has been unequivocally established, the role of specific variants in DLB pathogenesis is less clear, due to disparities between studies in classification of *GBA* variants (analysis restricted to GD variants or also including other rare variants), and differential coverage of the gene (genotyping or whole gene sequencing). Furthermore, some studies do not analyse the p.Glu365Lys variant, as it does not cause GD when homozygous and only reduces enzyme activity by approximately half [85]. Nevertheless, p.Glu365Lys is associated with PD, and is thought to explain the genome-wide signal at the *SYT11-GBA* locus [86]. This may also be the case for DLB, as the strongest association at the *GBA* locus was in strong LD with p.Glu365Lys (*D′* = 0.9, *R*^2^ = 0.78).

Mutations in *GBA* reduce activity of its lysosomal enzyme, β-glucocerebrosidase (Gcase), and Gcase activity has been shown to be reduced in sporadic PD brains without *GBA* mutations, due to decreased amount of protein [87]. Interestingly, Gcase deficiency can be detected in the CSF of PD patients, irrespective of mutation carrier status [88], providing promise for use as a biomarker in PD. It is hypothesized that decreased Gcase activity leads to impaired degradation of alpha-synuclein within the lysosome, resulting in its accumulation. In turn, increased alpha-synuclein perturbs Gcase transport to lysosome, therefore furthering lysosomal dysfunction [89]. Thus far, most studies have analysed Gcase deficiency in PD, and it is not known whether the same occurs in DLB. Nevertheless, Gcase was also significantly reduced in DLB CSF in a small study comparing DLB, AD, FTD and controls [90], suggesting its potential use as a biomarker for disease.

## GWAS

The first DLB GWAS was conducted in late 2017, and incorporated a total of 1743 DLB patients (1324 of which had autopsy supported diagnosis) and 4454 controls [16••]. Genotyping data was enriched with SNPs imputed from the haplotype reference consortium, to allow detection of lower frequency variants. *SNCA*, *GBA* and *APOE* loci were significantly associated with DLB in the discovery and replication phases, as well as the meta-analysis of both stages. In the discovery phase, two other loci were genome-wide significant: *BCL7C/STX1B* (OR 0.74, 0.67–0.82; *p* = 3.30  ×  10^−9^) and *GABRB3* (OR 1.34, 1.21–1.48; *p* = 2.62  ×  10^−8^). However, the *GABRB3* signal did not maintain significant association when restricting analysis to pathologically diagnosed samples (*p* = 1.21  ×  10^−7^). Loci with *p* < 5  ×  10^−6^ were genotyped in the replication phase, and *CNTN1*, a locus that reached suggestive association in the discovery phase (OR 1.58, 1.32–1.88; *p* = 4.32  ×  10^−7^, pathologically diagnosed cases only), was significantly associated in the replication phase (*p* < 0.05). As *CNTN1* lies < 500,000 bp away from the *LRRK2* locus, samples that harboured the p.Gly2019Ser *LRRK2* variant were removed from analysis, without significant effect on the association at *CNTN1*. However, whether other *LRRK2* variants may mediate the association cannot be ruled out. *BCL7C/STX1B* was genome-wide significant in the meta-analysis; however, this was mainly driven by the discovery phase, and requires further replication. Despite the identification of interesting candidates, replication of novel associations is required, and further GWA studies with larger cohorts are warranted. Heritability was shown to be higher than expected given chromosome size on chromosomes 19, 5, 6, 7 and 13 but apart from chromosome 19, no genome-wide significant variants were identified, suggesting that, perhaps, other DLB genes may be present on these chromosomes.

## Studies in Familial Forms of DLB

Although not particularly common, several DLB families have been studied, but these have failed to shed much light on the genetics of the disease. Only a small proportion of patients within these families underwent genetic analysis, and if performed, it was usually minimal with very few cases assessed at the exome or genome levels. As mentioned above, variation in *SNCA* sequence or dosage has been identified in families as the cause of mixed PD and DLB phenotypes. Some DLB patients have a family history of neurodegenerative disease, but not necessarily of DLB, with family members being diagnosed with AD or PD. Alzheimer families with mutations in *APP*, *PSEN1* and *PSEN2* have been described with phenotypes of mixed parkinsonism and dementia suggestive of DLB [91–93], and extensive Lewy body pathology has also been found in Alzheimer’s families with these mutations [94, 95], suggestive of a possible mechanistic link. Indeed, DLB cases frequently present Aβ pathology at autopsy [96], and it has been suggested that Aβ accumulation can trigger Lewy body disease [97]. A recent review by Vergouw and colleagues [98] provides a summary of familial studies in DLB. The most promising study to identify a novel DLB gene relied on linkage analysis in a DLB family which identified a locus on chromosome 2q35-q36 [11]; however, no gene was subsequently identified [99]. This could be because the variant was not detectable with current technology or, perhaps, the linkage results were misleading.

As our knowledge of the disease progresses, the diagnostic criteria for DLB are updated (Fig. 1b) to provide increasing sensitivity and specificity for accurate diagnoses [1••]. It is also worth considering whether previous reports in DLB families, which occurred when the first consensus criteria were in use, have patients that would meet current diagnostic criteria for DLB. The lack of autopsy data in some of these studies makes a reliable diagnosis difficult. Moving forward, genetic analysis of multiple family members with well characterized clinical and autopsy data that meet more recent diagnostic criteria for DLB, will be paramount in identifying novel disease-causing genes.

## Genes Causative of Other Neurodegenerative Diseases

As DLB may clinically and pathologically resemble Alzheimer’s or Parkinson’s diseases, speculation as to whether AD- or PD-causing genes may also be involved in the pathogenesis of DLB prompted the study of these genes in small cohorts of mainly sporadic DLB cases. However, due to the phenotypic similarities between diseases, it is still unclear whether the mutations identified play a role in DLB or simply occur in misdiagnosed cases. This issue is complicated further by the heterogeneity of phenotype that can be associated with some of these mutations [41, 46–48, 91, 93, 100].

Mutations that are established to be pathogenic in *APP* and *PSEN1* were found in either a clinical DLB case [29]; or in pathologically confirmed DLB cases, of which 69% of the cohort also met pathological criteria for Alzheimer’s disease [27]. Furthermore, studies of genes that cause other neurodegenerative diseases have identified variants in DLB patients that have been previously reported, but are of unknown pathogenic consequence in genes such as *CHMP2B*, *SQSTM1*, *PSEN2* [30] and *GRN* [29]. Novel variants in *MAPT* [29] and multiple variants of uncertain significance have also been reported. A compound heterozygous mutation in *PARK2* was identified in a DLB patient [30]; however it is unclear whether the data was phased. Rare variants in *GRN* were hypothesized to be associated with DLB, albeit in a very small study of 58 DLB cases and 380 controls [32]. Nevertheless, if the reported mutation carriers do in fact have DLB, mutations in genes known to cause Mendelian forms of other neurodegenerative diseases only occur in a small proportion of DLB cases.

## Other Findings

The *MAPT* H1G sub-haplotype was associated with clinical DLB; however, the association was attenuated when pathologically diagnosed samples were included in analysis [24], weakening evidence for a role specific to DLB. The H1 haplotype may be associated with more severe alpha-synuclein deposition, as suggested in a small study of 22 DLB brains [101]. *MAPT* p.Ala152Thr has been proposed to be associated with DLB in a clinical DLB cohort [25]. This variant is also associated with AD and FTD-spectrum disorders [102], but not PD [25, 102]. However, the *MAPT* locus showed no evidence of association in the DLB GWAS [16••], and this is of interest as the *MAPT* locus is a highly significant result in PD, reaching genome-wide significance even in smaller studies of approximately the same number of cases as the DLB GWAS [103]. Therefore, there is limited convincing evidence for a role of *MAPT* variation in DLB, and further studies of risk associated haplotypes in larger cohorts are needed.

Variants in the *TREM2* gene confer a risk for the development of AD similar to that associated to one ε4 allele of *APOE,* an association mediated primarily by the p.Arg47His variant, but also by others, such as p.Arg62His. The p.Arg47His variant was not found to be associated with DLB [16••, 22]; however, in a small study of 58 DLB cases, p.Arg62His was nominally associated with DLB (uncorrected *p* value = 0.0024, OR = 3.2 [95% CI 1.7–27] [32], although this would not survive multiple-test correction. Again, further study is required to identify the role, if any, of *TREM2* in DLB.

Pathogenic hexanucleotide repeat expansions in the *C9ORF72* gene are the most common cause of FTD and/or ALS. Analysis of the expansion in neuropathologically diagnosed DLB patients found three cases with either 32 or 33 repeats, in a combined total of 1562 patients [17, 19, 104]; whereas studies of clinically diagnosed DLB patients have identified two patients with > 30 repeats [18, 19, 105]. This suggests that repeat expansions in *C9ORF72* are not a common cause of DLB.

An increased frequency of the *LRRK2* p.Gly2019Ser variant was seen in DLB compared to controls (0.0021 versus 0.0003, respectively) [16••]; however, this was not statistically assessed given its low frequency. This variant has previously been seen in a clinical DLB case [26], and in two Ashkenazi Jewish individuals with DLB [80], demonstrating a low frequency in this disease. Where studied, no other pathogenic *LRRK2* mutations were found in DLB cases [27, 29, 30], and no *LRRK2* variants were significantly associated with DLB [26]. Whilst *LRRK2* genetic variation does not seem to occur often in DLB, it is difficult to distinguish between PD, PDD and DLB and thus assess the contribution of *LRRK2* specifically to DLB [106]. *LRRK2* mutation carriers in PD have a lower rate of dementia [107], perhaps providing further evidence against a role in DLB.

The apparent lack of association for other strong AD and PD risk loci, such as *TREM2*, *MAPT*, *CLU*, *PICALM*, and *BIN1* may hint at distinct genetic features for DLB, or may be attributed to insufficient power to detect associations.

## Requirements for Future DLB Genetic Studies

Genetic research in DLB is only just beginning to come together, providing hope for future characterization of the genetic architecture of the disease. In order to identify additional genes implicated in DLB, it will be imperative to study more individuals with the disease. This will require collaborative approaches in order to increase cohort numbers and more studies that are focused on replicating results. As well as generating genetic data, it is important to collect detailed clinical and pathological data on patients studied.

There is a clear need for more unbiased genetic studies (genome- or exome-wide). The majority of genetic studies in DLB thus far have been hypothesis based, largely trying to identify candidate genes (Table 1). Table 1 also highlights the fact that some DLB samples have been used in multiple genetic studies, an event that should be made clear and that can severely bias results, certainly for a disease where sample collections are small.

Large-scale genetics studies in neurodegenerative diseases have been dominated by European and American cohorts. The study of patients from other populations could allow the discovery of population-specific, predisposing variants, which in turn may provide novel insights into the biological processes that occur in disease. DLB research in Japan has been invaluable for furthering our understanding of the disease. For instance, the earliest identification of DLB patients [108], and the study of [(123)I]MIBG myocardial scintigraphy to distinguish DLB [109], were first proposed by Japanese scientists. Detailed, large-scale genetic analyses of these patients could be transformative for the field.

## Conclusion

Understanding the genetic bases of a disease can allow us to identify pathways and mechanisms involved in the disease pathobiology. An example of this is in AD, where the identification of *APP*, *PSEN1* and *PSEN2* mutations were crucial for the development of the amyloid cascade hypothesis. Furthermore, genetics may be able to help us tease apart PD, AD, DLB and PDD using molecular data. As diagnostic criteria [1••] and understanding of the disease improves [110–112], we will be able to have a better understanding of the biological processes underlying DLB, and this may lead to the identification of disease-specific therapeutic targets.

Next-generation sequencing technology has revolutionized genetic analysis, and in combination with large-scale databases of genetic variation such as gnomAD or ExAC [113], has allowed us to have a better understanding of genetic variation in humans. This knowledge is going to improve as datasets increase—the 1000 Genomes Project, which began in 2008, included whole genome data from 2504 individuals [114], yet current databases such as ExAC and gnomAD [113], now provide data from 60,706 exomes or 123,136 exomes and 15,496 genomes, respectively. Although these types of datasets will include information from individuals who will suffer from neurodegenerative disease, they have been invaluable in informing the evaluation of potentially pathogenic variants [115]. Furthermore, improved interpretation of GWAS results will enable genetic variants to be linked to a functional role in disease risk [116]. In addition, polygenic risk scores can be used to calculate an individual’s lifetime risk for disease based on their genetic profile, a process that has already been implemented in Alzheimer’s and Parkinson’s diseases [117, 118], and that may allow for identification of presymptomatic individuals.

The role for genetics in human biology is multifaceted and complex, and thus we need to integrate DNA sequencing, with RNA and protein expression, tissue and cell-specific expression and epigenetic analyses in order to reveal as complete a picture as possible. The overall objective is to understand the pathways that are perturbed in disease and that should be therapeutically targeted. Therapies will likely need to be administered prior to clinical disease onset, and therefore must target the prodrome. Genetic studies may allow us to identify those particularly susceptible for the development of the disorder, by utilizing polygenic risk scores or causative mutations.

Moreover, genetics has informed the use of biomarker: by associating genes with disease, there is the potential to analyse the proteins encoded by those genes in patients in order to aid diagnosis. For example, it has been suggested that Gcase [90] or alpha-synuclein [54] levels may be altered in the CSF of DLB patients. A recent study identified phosphorylated alpha-synuclein in skin biopsies of DLB patients, which was absent in controls, or those with an alternative diagnosis of dementia [119]. Although requiring replication, phosphorylated alpha-synuclein in skin was suggested to contribute to autonomic dysfunction and may provide an easy and relatively inexpensive biomarker for DLB [120].

Although DLB genetic research is still only in its beginning, interest in this area has increased, resulting in the identification of several genes that are involved in the disease. This is the first step to obtain a complete picture of the genetic architecture of DLB. As we get closer to this stage, we will be able to better understand disease pathogenesis and to nominate candidate disease-specific therapeutic targets, which will enable us to slow, or even halt, this devastating disease.
